# The Effectiveness of Silver Nanoparticles Mixed with Calcium Hydroxide against *Candida albicans*: An Ex Vivo Analysis

**DOI:** 10.3390/microorganisms12020289

**Published:** 2024-01-29

**Authors:** Maha Alghofaily, Jood Alfraih, Aljohara Alsaud, Norah Almazrua, Terrence S. Sumague, Sayed H. Auda, Fahd Alsalleeh

**Affiliations:** 1 Restorative Dental Sciences, College of Dentistry, King Saud University, Riyadh 11461, Saudi Arabia; falsalleeh@ksu.edu.sa; 2 College of Dentistry, King Saud University, Riyadh 11461, Saudi Arabia; joodalfraih@gmail.com (J.A.); noura.almaz@gmail.com (N.A.); 3 Molecular and Cell Biology Laboratory, Prince Naif Bin AbdulAziz Health Research Center, College of Dentistry, King Saud University, Riyadh 11461, Saudi Arabia; tsumague@ksu.edu.sa; 4 Department of Pharmaceutics, College of Pharmacy, King Saud University, Riyadh 11461, Saudi Arabia; sauda@ksu.edu.sa

**Keywords:** endodontic therapy, *Candida albicans*, root canal medicaments, calcium hydroxide, chlorhexidine irrigation, nanotechnology, silver nanoparticles

## Abstract

Introduction: The purpose of this study was to assess the antifungal activity of silver nanoparticles (AgNPs) in combination with calcium hydroxide (Ca(OH)_2_) against *Candida albicans* (*C. albicans*). Methods: AgNPs was mixed with pure Ca(OH)_2_ powder in an aqueous base. A standard suspension (1 × 10^8^ bacterial cells/mL) of *C. albicans* was prepared in a 96-well plate and incubated on shaker at 37 °C in 100% humidity to allow fungal biofilm formation in infected dentin slices (*n* = 98). The minimum inhibitory concentration (MIC) and minimum fungicidal concentration (MFC) of AgNPs alone or with Ca(OH)_2_ were determined. The samples were separately placed in 24-well tissue culture plates and divided into three experimental groups (0.03, 0.04, and 0.06) and three control groups; negative (saline) and positive chlorhexidine gel and Ca(OH)_2_. Quantitative measurements of fungal activity by XTT colorimetric assay and qualitative measurements using confocal laser microscopy and scanning electron microscopy were performed. Results: The cell viability of *C. albicans* in the experimental groups was significantly reduced compared to the negative control group. The combination of (AgNPs (0.04%) and Ca(OH)_2_) was the most potent against *C. albicans*. Conclusions: The findings demonstrated that combining silver nanoparticles with Ca(OH)_2_ was more effective against *C. albicans* biofilm compared to Ca(OH)_2_ alone, suggesting a combing effect.

## 1. Introduction

The oral cavity offers a particularly complex environment for fungal colonization due to its anatomical composition, which includes soft mucosal and mineralized hard tooth tissues, as well as the presence of abiotic surfaces such as dentures and implants [1]. *Candida albicans* (*C. albicans*) is most frequent commensal yeast of the microbiome, occurring in 30–45% of healthy adults [2], and is periodically pathogenic in the oral cavity [3]. *C. albicans* form and live within a biofilm matrix composed of exopolysaccharides, proteins, and nucleic acids that protect them from the environment and immune system [4,5]. Biofilm formation, leading to immune evasion and immune modulation of the host defense, is considered a key virulence factor of *C. albicans* [4,5,6]. Forming a biofilm can confer protection to the community of *C. albicans* against antimicrobial agents, in contrast to those in a non-adherent state, such as planktonic cells [7]. There is a growing recognition of the tooth surface as a significant habitat for Candida colonization and the production of dental biofilm alongside bacterial species [8]. The presence of the smear layer enhances the adherence of *C. albicans* to human dentin [9,10], perhaps because of the presence of exposed dentinal collagen and calcium ions. Candida can attach itself to collagen types I and IV [11].

The Candida microbiome significantly influences the progression of dental caries and the subsequent complications in endodontics [12,13]. The pathogenesis of apical periodontitis (AP) in endodontic diseases is driven primarily by the cumulative pathogenicity of a multispecies microbial community and their byproducts operating as a unit of “microbiota” [14,15]. Bacteria are the most abundant and influential microorganisms in endodontic infections; however, fungi, archaea, and viruses have also been discovered in connection with apical periodontitis [16]. The prevalence of fungi in main and secondary infections, as determined by culture-based research, was 6.3% and 7.5%, respectively. However, when molecular studies were conducted, the prevalence increased to 12.5% and 16.0% for primary and secondary infections, respectively [2]. *C. albicans* is the most frequently isolated fungi from endodontic lesions [17]. A pure culture of *C. albicans* has been found to be the causative factor of an acute apical abscess [18]. The presence of *C. albicans* is more pronounced in the root canal systems of teeth with periapical lesions [19].

Several studies have evaluated the effectiveness of different intracanal medications against *C. albicans*, including, calcium hydroxide (Ca[OH]_2_) paste and chlorhexidine (CHX) [20]. Calcium hydroxide and CHX are the two most often employed intracanal medicaments. The first medicament regarded an excellent antibacterial agent due to its high pH value. Additionally, it can denature proteins by releasing hydroxyl ions. Hence, Ca(OH)_2_ can efficiently eradicate the predominant pathogens in endodontics when employed as intracanal medicaments [21]. However, some investigations have raised doubts about the efficacy of Ca(OH)_2_ against specific pathogen including e faecalis [22,23], *C. albicans* [24]. It has been shown that *C. albicans* were resistant to Ca(OH)_2_ and additional medications were required to increase the effectiveness of anti-fungal activity [25]. It was reported that mixing Ca(OH)_2_ with chlorhexidine was more effective in eliminating *C. albicans* [26].

Nanotechnology has gained popularity in the fields of medicine and dentistry [27]. Nanoparticles are cationic particles synthesized from silver, copper oxide, zinc oxide, and other powdered particles that adhere to negatively charged dentin surfaces to prevent biofilm formation [28,29]. Historically, silver was employed as an oral antibacterial treatment prior to the advent of antibiotics [30], as well as to prevent wound contamination in burn patients [31]. Nowadays, it continues to be utilized in pharmacology as a carrier for targeted drug delivery. Silver containing gels possess antimicrobial properties against the tested microorganisms, including *C. albicans* [32]. Silver nanoparticles (AgNPs) alone or in combination with other antibiotics or Ca(OH)_2_ [33] are a promising alternative to currently available intracanal medications owing to their strong antibacterial properties [29,34]. Nanoparticles have been used as irrigants [35,36,37], intracanal medicaments [33,37,38,39], and additives in sealers or restorative materials [40,41,42,43] in addition to their utilization in periodontics as well as their application in dental implantology and dental prosthetics [44]. Silver nanoparticles (AgNPs) demonstrate bactericidal properties by breaking the cellular membrane, inhibiting metabolic enzymes, and generating reactive oxygen species (ROS) [45]. Previous research reported the potential antibacterial efficacy of AgNPs when combined with Ca(OH)_2_ [33,39,46]. Therefore, this study aimed to assess the antifungal activity of a combination of AgNPs and Ca(OH)_2_ against *C. albicans*. The null hypothesis was the combination of silver nanoparticles and calcium hydroxide had no antifungal effect against *C. albicans*.

## 2. Materials and Methods

### 2.1. Ethical Approval

The present study received ethical approval from the Institutional Review Board (approval no.: E-22-7221) at King Saud University, Riyadh, Saudi Arabia. An informed consent form was signed by the subjects to collect teeth samples.

### 2.2. Determination of the Minimum Antifungal Concentration

The study employed the serial broth dilution technique to ascertain the minimum inhibitory concentration (MIC) of various doses of AgNPs (ranging from 0.01% to 0.15%), both individually and in conjunction with Ca(OH)_2_, against the microorganism *C. albicans*. The minimum inhibitory concentration (MIC) was calculated in order to ascertain the optimal concentration of AgNPs that would yield the highest effectiveness based on previous studies protocol [37,47,48]. The test tubes were subjected to incubation at a temperature of 37 °C for a duration of 24 h under anaerobic conditions. Concentrations both below and above the chosen effective concentration were selected.

### 2.3. Determination of the Minimum Fungicidal Concentrations

A volume of 25 μL from each tube containing the solutions that demonstrated inhibition of visible fungal growth in the MIC experiment was aseptically transferred onto agar plates. The plates were subsequently incubated at a temperature of 37 °C for a duration of 48 h within an anaerobic jar, utilizing an anaerobic kit. The minimal fungicidal concentration (MFC) was determined as the lowest concentration on the agar plate that inhibited the growth of the majority of organism [47].

### 2.4. Sample Size Calculation and Sample Collection

The sample size was computed using G*Power 3.1.9.4 software. At a level of significance (a) = 0.05, an estimated effect size of 0.3, and with the power equal to 0.93; the total sample size was estimated to be 98, with 13 samples per experimental group. A diamond blade (MK-303) within a low-speed saw (IsoMet; Buehler, Lake Bluff, IL, USA) was used to section extracted single-canal teeth to obtain 98 radicular dentin samples (4 mm × 4 mm× 1 mm), as described in a previous study [49]. Samples with cracks or other abnormalities were excluded prior to commencing the experiment. The samples were subsequently sterilized using gamma radiation at a dose of 25 kGy. All experiments were performed in triplicate.

### 2.5. Preparation of AgNPs and Ca(OH)_2_

#### 2.5.1. Preparation of Silver Nanoparticle Liquefied Gel

The preparation was similar to previous work [46]. The amount of CMC (0.5%) was dispersed in the distilled water with gentle stirring (120 rpm) using a magnetic stirrer. Stirring was continued until no lumps were observed. The calculated amount of silver nanoparticles was dispersed in the prepared gel and stirred gently for one hour then put in a sonicator for 2 h. In order to have a homogeneous liquefied gel without entrapped air, the sample was left in a refrigerator (4 °C) for 4 h.

#### 2.5.2. Preparation of Silver Nano-Calcium Hydroxide Paste

Viscous vehicles (glycerine, polyethyleneglycol, propyleneglycol) are water-soluble substances that release Ca++ and OH- ions more slowly for extended periods. These should be used for redressing, because the paste may remain in the root canal for a longer period [50]. The silver nano-calcium hydroxide paste was prepared by livegation of silver nano and calcium hydroxide powder with a viscous aqueous vehicle (propyleneglycol:glycerine 1:1) to produce a consistency similar to that of toothpaste.

### 2.6. Candida Strain and Media and Biofilm Formation

The *C. albicans* wild-type strain (CA42) (ATCC^®^ 10231™) was cultivated under aerobic conditions in yeast nitrogen base (YNB) medium (Difco Laboratories, Detroit, MI, USA) on a newly prepared Sabouraud Dextrose Agar plate (Difco Laboratories, Detroit, MI, USA). The plate was placed in an incubator and kept at a temperature of 37 °C for a duration of 24 h at a shaker at 60 rpm (model classic C25, New Brunswick Scientific, Edison, NJ, USA). The cells were collected and rinsed three times with 0.15 M phosphate-buffered saline (Gibco PBS; pH 7.4, without calcium and magnesium ions, Life Technologies, Grand Island, NY, USA). The cells were suspended in 10 mL of PBS, enumerated using a hematocytometer, and utilized within a 24 h timeframe.

Dentin samples were placed onto 96-well tissue culture plates. They were immersed in a *C. albicans* suspension containing 10^5^ cells/mL, which yielded 20,000 cells per well. Following the allocation of the samples into their designated well plates, each well was supplemented with 10 µL of *C. albicans* containing 1 × 10^8^ cells/mL, along with 1 mL of YPD medium. The well plates were then incubated at a temperature of 37 °C in an atmosphere containing 5% CO_2_ for 14 days. This incubation period was intended to create an optimal environment conducive to the maturation phase of the biofilm [51]. The culture medium was changed biweekly.

### 2.7. Treatment of Infected Specimens

The samples underwent a gentle rinsing process using sterile phosphate-buffered saline (PBS) in order to eliminate the culture medium and non-adherent Candida.

Ninety-eight dentinal slices were allocated randomly into three experimental groups and three control groups, and subsequently transplanted to 24-well tissue-culture plates. A total of 1.0 mL of the designated medication was introduced into each well utilizing a syringe, and then subjected to incubation at a temperature of 37 °C for a period of 7 days.

1. Negative control: non-infected dentin samples.
2. Positive control: infected dentin samples
3. Ca(OH)_2_ group: Ca(OH)_2_ alone (35%)
4. Chlorohexidine: 2% of Chlorhexidine Antibacterial Viscous Solution (Consepsis, UltraDent, South Jordan, UT, USA)
5. Experimental groups: the three best concentrations selected from MIC and MFC as follows:

0.06% AgNPs plus Ca(OH)_2_

0.04% AgNPs plus Ca(OH)_2_

0.03% AgNPs plus Ca(OH)_2_

**Figure 1:**
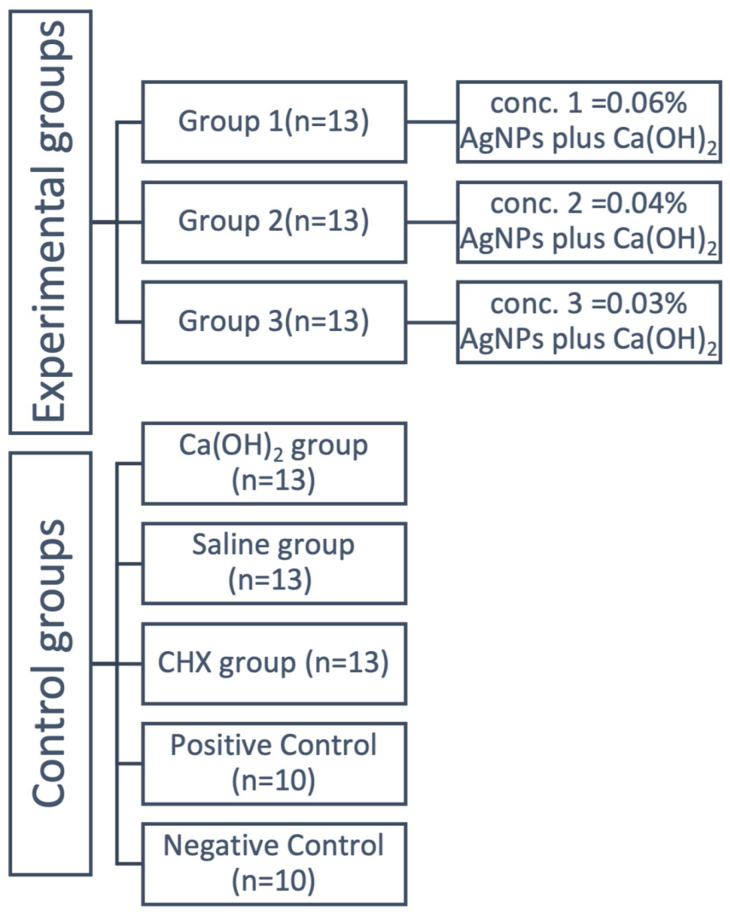
Flowchart of the selected experimental and control groups.

The experimental groups that were chosen for the study, along with the control group, are displayed in Figure 1.

### 2.8. Antifungal Activity

The antifungal activity was measured using two methods: quantitative measurements of fungal activity via XTT colorimetric assay and qualitative measurements using confocal laser microscopy and SEM.

### 2.9. XTT

XTT colorimetric assay [2,3-bis(2-methyloxy-4-nitro-5-sulfo-phenyl)-2H-tetrazolium-5-carboxanilide] was used to quantitatively determine the metabolic activity of *C. albicans* 7 days after the application of the medicament. The treated samples were removed from the wells and gently rinsed with PBS. The samples were then placed in a 96-well plate and 4 µL of menadione mixed with the XTT powder was added to each well to attain a volume of 50 μL. The 96- and 24-well plates with the negative and positive controls that contained broth only were wrapped in foil and placed in the shaker for 5 min. The samples were then incubated at 37 °C for 5 h using incubator shaker (New Brunswick Scientific, Excella E24 Incubator Shaker Series, Eppendorf, Hamburg, Germany) and placed into the microplate reader (BioTek®, Winooski, VT, USA). The samples were scraped vigorously after the first reading and placed into the microplate reader for a second reading. The remaining menadione and XTT powder liquid was placed in Eppendorf tubes and centrifuged for 5 min at 3500× *g* and placed into the microplate reader to obtain the final reading. The readings were performed in triplicate, with each value representing the mean absorbance (optical density) measured at a wavelength of 590 nm.

### 2.10. Scanning Electron Microscopy

Selected samples from each medicament group underwent SEM analysis at the end of each observation period. The treated samples were rinsed gently with PBS, transferred to 24-well plates, and dried for 3 days. The specimens were then sputter-coated with gold and analyzed using SEM (Jeol, Akishima, Japan JDM-6610LV). Micrographs were obtained from random areas of each specimen at 5000× magnification and observed digitally.

### 2.11. Confocal Laser Microscopy

Selected samples from each medicament group underwent confocal microscopy to evaluate the antifungal activity among the groups and observe the pattern of microbial colonization as described in a previous study [52]. The treated samples were rinsed with PBS and stained with a live/dead BacLight Bacterial Viability Kit (Molecular Probes, Eugene, OR, USA) and subsequently examined using confocal laser scanning microscopy (CLSM) (Nikon C2^1^ system, Nikon Instruments Inc., Melville, NY, USA).

### 2.12. Measurement of pH

A pH meter (Model DM 22, Digimed, São Paulo, SP, Brazil) was used to determine the pH of the selected concentrations.

### 2.13. Statistical Analysis

Statistical analysis was performed using IBM SPSS Statistics software (version 28). Data obtained regarding the antifungal activity were analyzed using one-way analysis of variance. Tukey’s post hoc test was used to compare the differences between groups at the same time points. The level of significance was set at 0.05, and *p* < 0.05 was considered statistically significant.

## 3. Results

### 3.1. Determination of the Minimum Antifungal Concentration

Table 1 presents the results of the MIC experiments. Turbidity was observed in some test tubes after the incubation period, indicating the growth of *C. albicans.* The MIC and MFC values of AgNPs alone or in combination with Ca(OH)_2_ were used for further analyses. Higher concentrations of AgNPs for both preparations were also analyzed. The three most effective concentrations of AgNPs were 0.03, 0.04, and 0.06.

### 3.2. Reduced Proliferation of C. albicans after Medicaments

**Figure 2:**
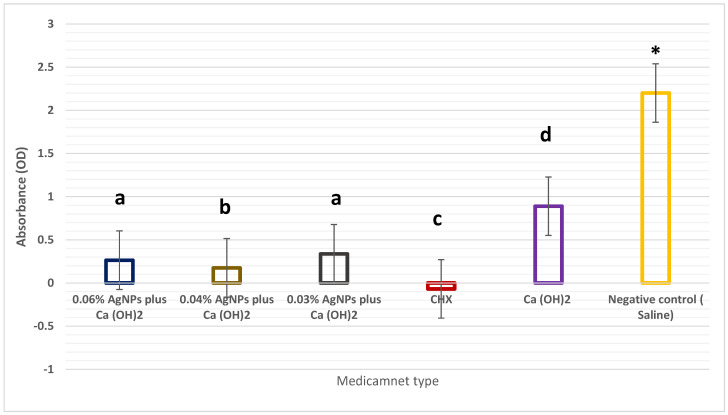
XTT generation illustrates the impact of several medications on the dentin slices containing *C. albicans* biofilm incubated for a period of 7 days. The presence of various letters on bars denotes statistical significance (*p* < 0.05), while the same letters do not show any statistically significance. The asterisk (*) is used to denote statistical significance (*p* < 0.05).

Figure 2 presents the results of the XTT analysis. A significant reduction in fungal viability compared with that of the negative controls was observed in all experimental groups after 7 days (*p* < 0.05). A more substantial reduction in candida cells was observed after 7 days with AgNPs with concentrations of 0.04% and 0.06%. CHX exhibited the strongest antifungal effect against *C. albicans*. Table 2 presents the descriptive statistics.

### 3.3. Scanning Electron Microscopy Analysis

**Figure 3:**
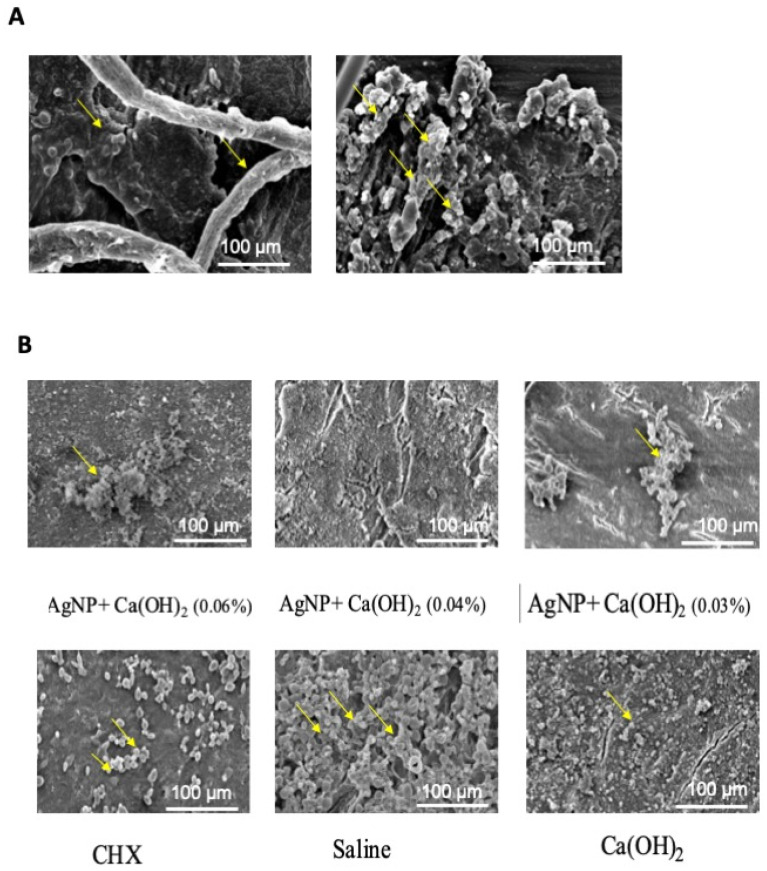
Scanning electron microscopic images of *C. albicans.* (**A**) images of 4 weeks *C. albicans* biofilm on dentin slices. Yellow arrows indicate the biofilm composed of hyphae. (**B**) Dentin slices were treated with experimental groups for seven days: AgNPs (0.06%), AgNPs (0.04%), AgNPs (0.03%) and control groups: saline as a negative control, and CHX and Ca(OH)_2_ as positive controls. SEM: Scanning electron microscopy analysis was performed at a voltage of 10 kV and a magnification of 1000×. Yellow arrowheads indicate biofilms of *C. albicans*.

Micrographs of the positive specimens were acquired at 1000× magnification to confirm biofilm formation. Magnified images were acquired from random areas of each sample of the experimental groups and digitally observed. The invasion by yeast cells extended to the entire tubular pathway (Figure 3A). SEM analysis revealed that the AgNP treatment was effective in removing biofilms (Figure 3B). Slight tubule penetration was observed, albeit in some tubules. CHX treatment was also found to be effective in removing the biofilm.

### 3.4. Quantitative and Qualitative Measurements of Antifungal Activity Using Confocal Laser Microscopy

**Figure 4:**
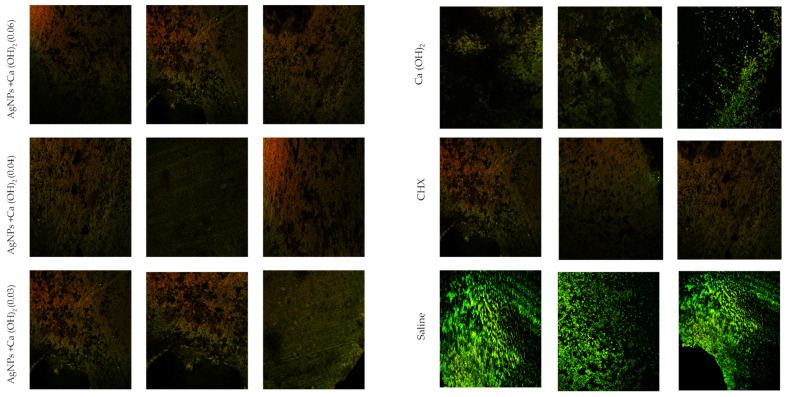
Confocal laser microscopy images of *C. albicans* infected dentin disks treated with different medicaments: AgNPs (0.06%), AgNPs (0.04%), AgNPs (0.03%) and control groups: saline as a negative control, and CHX and Ca(OH)_2_ as positive controls. Live cells seen in green, and dead cells seen in red.

The treated samples were rinsed in PBS and stained with live/dead stain for 30 min. Random areas of the samples were selected to undergo confocal laser microscopy. All infected specimens from the control group that were maintained in sterile saline solution for different periods of time yielded positive cultures. Disinfection of the dentin and elimination of a large number of *C. albicans* cells in the tubules was achieved in 7 days for the samples treated with Ca(OH)_2_/AgNPs or CHX. However, the Ca(OH)_2_ paste was found to be ineffective in disinfecting dentin, even after 1 week (Figure 4).

### 3.5. pH Evaluation

The measurements of the pH levels of AgNPs + Ca(OH)_2_ in Group 1 (0.06%), Group 2 (0.04%), and Group 3 (0.03%) is presented in Table 3.

## 4. Discussion

The present study tested the null hypothesis that combining AgNPs and Ca(OH)_2_ would not be effective against *C. albicans*. The antifungal efficacy of the positively charged paste containing a combination of AgNPs and Ca(OH)_2_ was evaluated 14 weeks after the formation of *C. albicans* on root dentin as demonstrated in previous studies [51,53]. In our study, the application of Ca(OH)_2_ alone yielded limited antifungal activity; however, the use of gels containing various concentrations of AgNPs (medicament) for 7 days resulted in significant destruction of the biofilm structure of *Candida* and a significant reduction in the number of viable cells in the biofilms. With the application of Ca(OH)_2_ alone, the most often utilized carriers are successful in delivering hydroxyl ions, without having any antibacterial properties. Therefore, the combination of medications and the vehicle (AgNPs) can result in additive or synergistic effects [54]. The suspension of silver nanoparticles proved to be the most efficient medium for delivering calcium hydroxide as an intracanal medicament against *Candida albicans* [55]. The efficacy of the addition AgNPs in a Ca(OH)_2_ mixture showed promising antibacterial properties against a wide range of microorganisms [24,39,46]. Their defense mechanism against bacteria was elucidated by the antibacterial activity of the AgNPs; their particles consistently emit silver ions, which can be employed as a tactic for eradicating microorganisms [56]. Silver has a positive charge, causing it to readily interact with negatively charged biomolecules like phosphorous and sulfur. These biomolecules are crucial constituents of the cell membrane, proteins, and DNA bases. This, in turn, destroys biofilms [57]. Previous reports showed the combination of SNP and Ca(OH)_2_ was found to be superior when compared to SNP and Ca(OH)_2_ used individually against *C. albicans* or in the eradication of Enterococcus faecalis (*E. faecalis)* [24,33]. Although the presence and growth of fungi in the root canal system has been reported [10,58,59], few studies have examined the effectiveness of antimicrobial agents *C. albicans* [17,24,25,60]. Unlike bacteria and yeasts, the study of the interactions between AgNPs and filamentous fungus (molds) is still developing. Other studies explored the mechanisms of antifungal efficacy via the disruption of the integrity of the membrane and cell components [61,62].

The use of CHX resulted in a significant reduction in fungal growth, indicating that it was the most effective agent among the experimental and Ca(OH)_2_ paste groups The antibiofilm effect with the use of CHX alone or in combination of Ca(OH)_2_ was previously reported against *C. albicans* [20,21,63]. Waltimo et al. demonstrated that a combination of Ca(OH)_2_ and CHX acetate was more effective against *C. albicans* than pure Ca(OH)_2_; however, this combination was less effective than CHX alone [25]. The pH of CHX was measured and assigned five values, which revealed that the antimicrobial activity of this medicament was pH-dependent, with an optimum range of 5.5–7.0. The antimicrobial effectiveness of this substance was significantly reduced as the pH increased [64]. CHX is effective because it has the ability to bind to negatively charged surfaces. It is released slowly from these surfaces, which allows it to continue its antibacterial action for several hours. This aforementioned process is called substantivity [65]. However, an important fact to be pointed out is that the combination of CHX with NaOCl produces an orange-brown precipitate, which forms a chemical smear layer. This layer covers the dentinal tubules and has the potential to disrupt the seal of the root filling. Also, this precipitate induces a change in the tooth’s color [66,67,68]. Although, this interaction also contributes to the antibacterial properties, it exhibits cytotoxic characteristics [69].

In our study, a substantial reduction in candida cells was observed after 7 days with AgNPs of concentrations 0.04% and 0.06% unlike in Ca(OH)_2_ alone, which showed limited activity against *C. albicans*. Ca(OH)_2_ exhibits broad-spectrum antibacterial properties against prevalent endodontic infections, but its efficacy is diminished when targeting *E. faecalis* and *C. albicans*. [25,60,70]. This effect may be attributed to the fact that dentin and the biofilm matrix of *C. albicans*, similar to that of *E. faecalis*, neutralize the high alkalinity of Ca(OH)_2_, which collectively contributes to the diminished antibacterial activity of Ca(OH)_2_ [4,71]. The measurement of the pH level revealed that 0.06%, 0.04%, and 0.03% of AgNPs + Ca(OH)_2_ were related to high alkalinity, similar to Ca(OH)_2_. Alkaline intracanal medicaments and irrigants with higher pH levels can eradicate biofilm-forming microorganisms; however, the alkalinity of the saturated Ca(OH)_2_ solution may not have any effect on *C. albicans* [72]. Although the antimicrobial mechanism of AgNPs is not fully understood, it has been proposed that these particles provoke the production of reactive oxygen species, cell membrane disruption, mitochondrial damage, and DNA damage when they come in contact with the cells [73].

A large amount of research has been conducted to assess the effectiveness of the amalgamation of AgNPs and Ca(OH)_2_ in combating diverse bacteria. The study conducted by Afkhami et al. provided evidence that the combination of Ca(OH)_2_ and AgNPs led to a notable reduction in colony count following a one-week period of exposure [39]. Zhang et al. conducted a study using plate culture count and crystal violet biofilm assay to compare the inhibitory effect of a combination of Ca(OH)_2_ and AgNPs on *E. faecalis* biofilms with that of AgNPs or Ca(OH)_2_ alone at 1 and 7 days [74]. SEM analysis was used to visualize the morphology and structure of the biofilms. The findings of the present study are consistent with those of a previous study, which demonstrated that AgNPs are potent inhibitors of both *E. faecalis* and *C. albicans* biofilm formation when combined with Ca(OH)_2_ [24]. SEM analysis findings were consistent with an overall loss of biofilm structure, mostly due to the disruption of the outer cell membrane or wall and inhibition of filamentation [71].

## 5. Conclusions

Knowledge of the mechanisms underlying the effect of AgNPs + Ca(OH)_2_ on *C. albicans* and other fungi can help optimize endodontic therapy and increase the success rate of treatment. Within the limitations of this study, it can be concluded that a combination of Ca(OH)_2_ and 0.04% AgNPs showed the most effective antibiofilm activity against *C. albicans* biofilm. Additional in vivo clinical studies must be conducted, thus progressing the field towards possible therapeutic use.

## Figures and Tables

**Table 1:** Minimum inhibitory concentration (turbidity) and minimum antifungal concentration (MIC) (Agar plate).

Concentrations	0.15%	0.1	0.06	0.04	0.03	0.02	0.01	Vehicle
AgNPs alone Turbidity								
	_	_	_	_	+	+	+	+
Agar growth	_	_	_	_	+	+	+	+
AgNPs plus Ca (OH)_2_ Turbidity								
	_	_	_	_	_	+	+	+
Agar growth	_	_	_	_	_	+	+	+

The sign (+ and _) represents growth and no growth of *C. albicans*.

**Table 2:** Means of the 2-methoxy-4-nitro-5-sulfo- phenyl)-2H-tetrazolium-5-carboxanilide values for the positive control groups and the experimental groups after contact with the experimental medications for 7 days compared to the negative control group.

Group	M ± SD	95% Confidence Interval
0.06% AgNPs plus Ca(OH)_2_	0.264 ± 0.043	0.156 to 0.372
0.04% AgNPs plus Ca(OH)_2_	0.175 ± 0.030	0.101 to 0.249
0.03% AgNPs plus Ca(OH)_2_	0.337 ± 0.117	0.046 to 0.627
CHX	−0.068 ± 0.011	−0.094 to −0.041
Ca(OH)_2_	0.889 ± 0.665	0.763 to 2.542
Negative control	2.200 ± 0.379	1.258 to 3.143

**Table 3:** Measurements of the pH levels of AgNPs + Ca(OH)_2_ in Group 1 (0.06%), Group 2 (0.04%), and Group 3 (0.03%), Ca(OH)_2_ alone, and CHX.

Group	Silver-Nano Concentration (%)	Ca(OH)_2_ Concentration (%)	PH Level
0.06% AgNPs plus Ca(OH)_2_	**0.06**	**35**	**11.18**
0.04% AgNPs plus Ca(OH)_2_	**0.04**	**35**	**11.19**
0.03% AgNPs plus Ca(OH)_2_	**0.02**	**35**	**11.21**
Ca(OH)_2_		**35**	**11.44**
CHX			**5**

## Data Availability

The data presented in this study are available on request from the corresponding author.
